# Draft Genome Sequence of the Rice Kernel Smut *Tilletia horrida* Strain QB-1

**DOI:** 10.1128/genomeA.00621-15

**Published:** 2015-06-25

**Authors:** Na Wang, Peng Ai, Yangfan Tang, Jing Zhang, Xiaojuan Dai, Ping Li, Aiping Zheng

**Affiliations:** a Rice Research Institute of Sichuan Agricultural University, Chengdu, China; b Key Laboratory of Southwest Crop Gene Resource and Genetic Improvement of Ministry of Education, Sichuan Agricultural University, Ya’an, China

## Abstract

*Tilletia horrida* is the most destructive fungal pathogen of rice (*Oryza sativa* L.). The 20,105,270-bp draft genome sequence of *T. horrida* strain QB-1 is reported here. Genes encoding proteins associated with key virulence factors were predicted, and this can provide information for understanding the pathogenic mechanisms in *T. horrida*.

## GENOME ANNOUNCEMENT

As the agent of the rice kernel smut, *Tilletia horrida* (also known as *Neovossia horrida*) is an important fungal pathogen of rice (*Oryza sativa L.*). It causes great yield losses in all rice-growing regions of the world. *T. horrida* belongs to the *Tilletia* genus, *Basidiomycotina Tilletiaceae* subdivision. In nature, *T. horrida* exists primarily as teliospores. The teliospores are widespread in the seeds of the host plant inside or outside and soil overwinter. Teliospores have a strong ability to resist adversity. Under natural conditions, they can survive for >1 year, and if they are stored inside seeds, they can survive for 3 years (1). During the infection stages, the key characteristic of the fungus is that it mainly infects rice grains from stigmas during early flowering to form a black sorus full of teliospores (2). After the introduction of hybrid rice in the 1970s, the prevalence of rice kernel smut further increased in paddy-irrigated rice areas and seriously influenced yield of rice and seed quality. Each year, rice kernel smut causes up to a 5% to 20% decrease and prevalence in diseased grains of as much as 40% to 60%. This disease has a wide distribution in rice-producing countries of Asia, the Americas, Africa, and most of the rice-growing areas of China. Despite the high worldwide rice yield losses caused by *T. horrida*, only limited information is available about its identification and chemical control. In this study, the *T. horrida* strain QB-1 isolate was sampled from the naturally seriously infected rice in China’s Sichuan Province in 2013. The genomic DNA was randomly interrupted and used to construct a pUC18 plasmid with a 180-bp inserted sequence. The genome was sequenced using Illumina Solexa GA II sequencing technology. The sequence was assembled achieved using the SOAP*denovo* assembly tool. The draft genome of *T. horrida* strain QB-1 is 20,105,270 bp and consists of 767 scaffolds, containing 1,713 contigs. The *N*_50_ sizes of the scaffold and contig are 75,652 bp and 31,277 bp, respectively. The G+C content of the genome is 55.86%. The genome of strain QB-1 was annotated using Augustus and showed 9,038 predicted genes. The proteins encoded by predicted genes of the strain was compared to proteins in the nonredundant (NR) database. The results show 4,650 proteins of the strain to be similar to proteins in the NR database, which accounts for 51.4% of all predicted proteins in the strain. The annotated draft genome sequence of *T. horrida* strain QB-1 made it possible to find genes encoding proteins associated with the key virulence factors, which might provide important information to properly understand the destructive pathogen *T. horrida*. The main objective of our study was to elucidate the possible molecular basis of host-pathogen interactions and the pathogenic mechanisms of the rice-infecting pathogen, which might provide knowledge for improving the yield of food crops in agriculture.

### Nucleotide sequence accession number.

This whole-genome shotgun project has been deposited in DDBJ/ENA/GenBank under the accession no. LAXH00000000.
